# Balancing Anodic Stability and Cathodic Kinetics in Practical Lithium‐Sulfur Batteries With Non‐fluorinated Weakly Solvating Solvents

**DOI:** 10.1002/advs.202417305

**Published:** 2025-07-02

**Authors:** Zhicheng Wang, Shixiao Weng, Haiyang Zhang, Liping Wang, Xu Yao, Haifeng Tu, Dan Huang, Suwan Lu, Lingwang Liu, Jiangyan Xue, Fengrui Zhang, Guan Wu, Jieyun Zheng, Qing Wang, Liquan Chen, Jingjing Xu, Hong Li, Xiaodong Wu

**Affiliations:** ^1^ Beijing Advanced Innovation Center for Materials Genome Engineering Key Laboratory for Renewable Energy Beijing Key Laboratory for New Energy Materials and Devices Institute of Physics Chinese Academy of Sciences Beijing 100190 China; ^2^ *i*‐lab Suzhou Institute of Nano‐Tech and Nano‐Bionics (SINANO) Chinese Academy of Sciences Suzhou 215123 China; ^3^ Tianmu Lake Institute of Advanced Energy Storage Technologies Co., Ltd. Liyang 213300 China; ^4^ School of Materials and Energy University of Electronic Science and Technology of China Chengdu 611731 China; ^5^ College of Material Science and Engineering Hohai University Changzhou 213000 China; ^6^ Department of Materials Science and Engineering National University of Singapore Singapore 117576 Singapore; ^7^ National Engineering Lab for Textile Fiber Materials & Processing Technology Zhejiang Sci‐Tech University Hangzhou 310018 China

**Keywords:** lithium sulfur batteries, long cycle life, shuttle effect, solvation structures, weakly solvating electrolytes

## Abstract

The performance of lithium‐sulfur (Li‐S) batteries is crucially affected by the anodic stability of Li‐metal and cathodic conversion kinetics of sulfur‐carbon (S/C) composites. Herein, a weakly solvating electrolyte (WSE) with moderate lithium polysulfides (LiPSs) solubility, consisting of 1 M lithium bis(trifluoromethane sulfonyl) imide (LiTFSI) in non‐fluorinated solvent of cyclopentyl methyl ether (CPME) and 1,3‐dioxolane (DOL) additive, is employed in Li‐S batteries to simultaneously achieve high anodic stability and appropriate cathodic kinetics. This WSE exhibits a good capability of suppressing LiPSs shuttling by forming an anions‐dominated Li^+^ solvation structure, effectively inducing a stable solid electrolyte interphase (SEI) to guarantee anodic stability of the Li‐metal anode. Additionally, the DOL additive in the WSE aids in forming a thin organic‐inorganic hybrid cathode electrolyte interphase (CEI) on the surface of S/Li_2_S particles, which maintains good conversion kinetics and suppresses dead S/Li_2_S growth in the S/C cathode. Consequently, Li‐S batteries with the WSE deliver a high initial capacity (≈1208 mAh g^−1^), a high average Coulombic efficiency (≈98.6%), and a high capacity retention rate (≈82.4%) over 200 cycles. Stable cycling performance over 100 cycles is also observed in the Li‐S pouch cell with the WSE even under harsh conditions.

## Graphical abstract

A weakly solvating electrolyte with non‐fluorinated cyclopentyl methyl ether solvent and 1,3‐dioxolane additive is employed in Li‐S batteries, which shows moderate lithium polysulfides solubility and improves the electrode interfacial stability. Therefore, appropriate cathodic kinetics and good anodic stability are both achieved in S/C||Li batteries, leading to good rate capability and stable cycling performance.

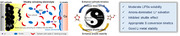

## 1 Introduction

Lithium (Li) metal, possessing ultrahigh theoretical specific capacity (3860 mAh g^−1^) and the lowest negative electrochemical potential (−3.04 V vs standard hydrogen electrode), is considered as the “Holy Grail” anode for next‐generation high‐energy‐density secondary batteries.^[^ ^1^, ^2^, ^3^ ^]^ Especially, Lithium‐sulfur (Li‐S) batteries, assembled with Li metal anode and sulfur‐carbon (S/C) composite cathode with naturally abundant and environmentally friendly S, have attracted extensive attentions owing to their high theoretical specific capacity and energy of 1675 mAh g^−1^ and 2600 Wh kg^−1^, respectively.^[^ ^4^, ^5^, ^6^, ^7^, ^8^ ^]^ However, there are several challenges that need to be addressed before Li‐S batteries can be widely deployed. One major issue is the shuttle effect, which occurs when lithium polysulfides (LiPSs, Li_2_S_4‐8_) dissolve in the traditional ether electrolyte.^[^ ^9^, ^10^ ^]^ This leads to inevitable parasitic reactions between LiPSs and Li metal, resulting in low Coulombic efficiency (CE) and rapid capacity decay of batteries.^[^ ^11^ ^]^ Another challenge is the large volumetric expansion and dendritic growth of Li metal during cycling, which limits the stability and safety of the batteries.^[^ ^12^, ^13^, ^14^ ^]^

Many efforts have been devoted to resolving the above issues. On the cathode side, physical hosts/barriers^[^ ^15^, ^16^, ^17^ ^]^ and chemical catalysis/adsorption^[^ ^18^, ^19^ ^]^ techniques have been employed to immobilize LiPSs. On the anode side, surface modification^[^ ^20^, ^21^ ^]^ and nanostructure design^[^ ^22^ ^]^ approaches have been developed to inhibit dendritic growth and volumetric expansion of Li metal. Different from separate modification and design of electrodes, rational design of electrolytes is an effective strategy to simultaneously suppress shuttle effect of LiPSs and improve the stability of Li metal anode.^[^ ^23^, ^24^, ^25^ ^]^ One kind of electrolytes is sparingly solvating electrolytes, such as high concentration electrolytes (HCEs),^[^ ^26^, ^27^, ^28^ ^]^ localized high concentration electrolytes (LHCEs),^[^ ^23^, ^29^, ^30^, ^31^ ^]^ and solvated ionic liquid electrolytes,^[^ ^32^, ^33^ ^]^ to realize minimal solubility of LiPSs and uniform Li deposition. For instance, Suo et al.^[^ ^26^ ^]^ developed a solvent‐in‐salt electrolyte with ultrahigh salt concentration, effectively inhibiting the LiPSs dissolution and improving the stability of Li metal anode in Li‐S batteries. Wang et al.^[^ ^23^ ^]^ incorporated an inert fluoroalkyl ether into HCE to create a LHCE, which reduced the electrolyte viscosity, improved wettability, and achieved high CE in Li‐S batteries. Nevertheless, these electrolytes with low LiPSs solubility severely deteriorate the cathodic kinetics, resulting in slow conversion of S species and inadequate utilization of active materials, finally leading to rapid capacity loss and increased polarization due to the continuous growth of dead S/Li_2_S.^[^ ^25^, ^34^ ^]^ Additionally, the high cost of large amounts of lithium salt or environmentally‐hazardous fluorinated ether hinders the affordability and widespread application of these electrolytes.

**Figure 1:**
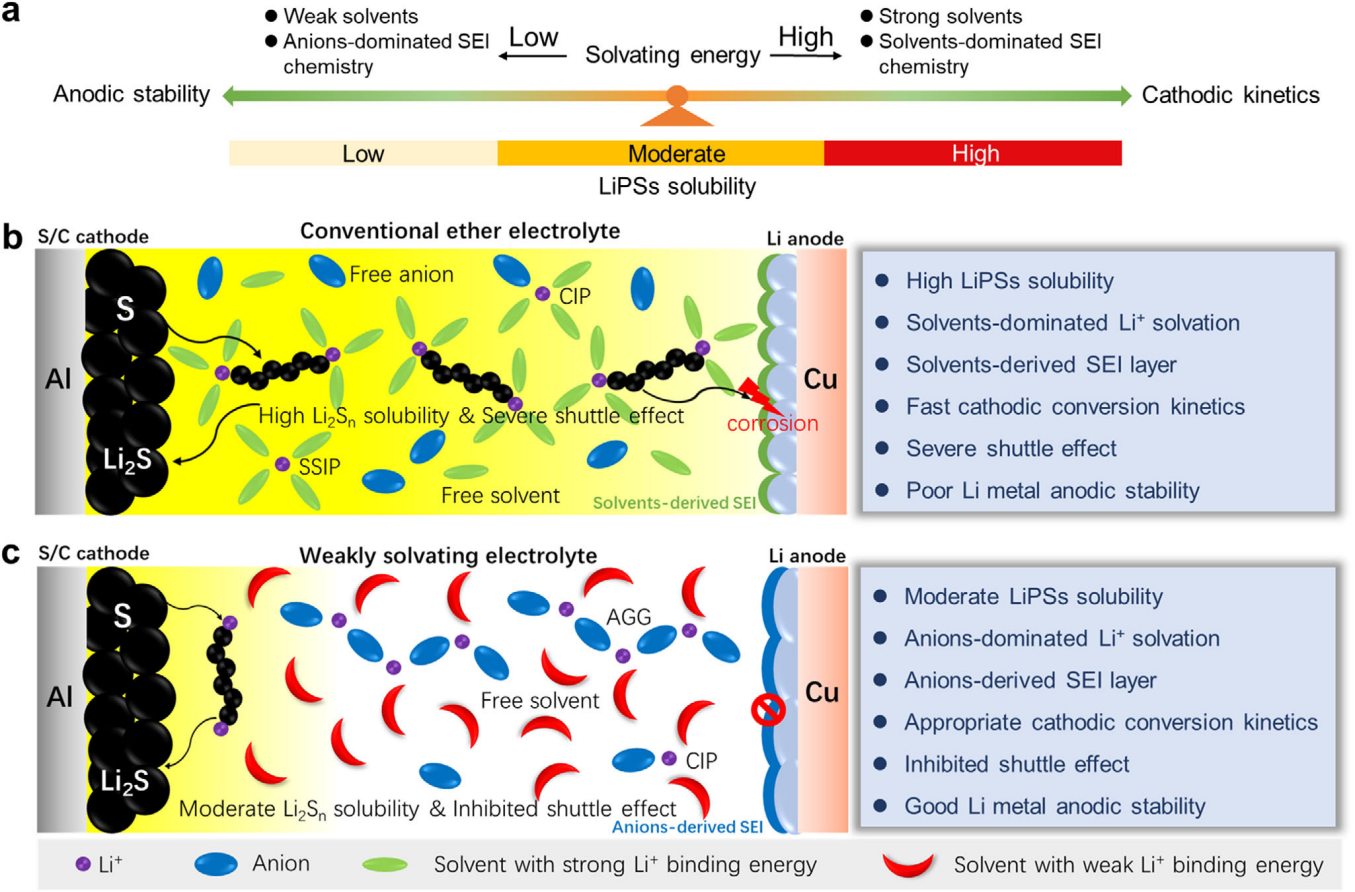
Conception of electrolyte design for high‐performance Li‐S batteries. a) Balancing cathodic kinetics and anodic stability by regulating solvating energy and LiPSs solubility of the electrolyte. Schematic diagram of interfacial chemistry and electrolyte solvation structures in Li‐S batteries with b) conventional ether electrolyte and c) weakly solvating electrolyte.

Most recently, electrolytes with moderate LiPSs solubility have been demonstrated to be promising approaches to achieve practical Li‐S batteries, because they can simultaneously mediate the conversion of S cathode and suppress LiPSs shuttling.^[^ ^35^ ^]^ In this study, fluorinated electrolytes, using standard salt concentration and solvents with different fluoridation degree, were prepared and employed in Li‐S batteries. Using these fluorinated electrolytes with moderate LiPSs solubility, Li‐S batteries can simultaneously achieve high discharge capacity, high CE and long‐calendar‐life. Therefore, as illustrated in **Figure** **1**a, from the perspective of solvent selection, the solvation energy of the solvent will significantly affect the electrolyte solvation structure and the solid electrolyte interphase (SEI) chemistry, and the solubility of LiPSs will also affect the cathodic kinetics and the anodic stability in the Li‐S battery. Only a good balance between LiPSs solubility and interfacial stability can achieve satisfactory cathodic kinetics and anodic stability in Li‐S battery. However, there are few reports on such suitable electrolyte with moderate LiPSs solubility by using low‐cost, environmental‐friendly and fluorine‐free solvents. At the same time, in‐depth study of the long‐term cycle stability and failure mechanism of Li‐S batteries in these electrolytes is crucial to further guide electrolyte optimization and promote its practical application in long‐cycle Li‐S batteries.

Here, we propose a weakly solvating electrolyte (WSE) with moderate LiPSs solubility for stable Li‐S batteries, by using 1 M lithium bis(trifluoromethane sulfonyl) imide (LiTFSI) salt in a non‐fluorinated cyclopentyl methyl ether (CPME)^[^ ^36^, ^37^ ^]^ solvent with 2wt% 1,3‐dioxolane (DOL) additive. The solvation structure of the WSE and its suppression effect on LiPSs shuttling are studied in this work, which is compared with the conventional ether electrolyte (1 M LiTFSI in dimethoxyethane (DME)/DOL by volume ratio of 1:1). As shown in Figure 1b, a DME solvent‐dominated Li^+^ solvation structure (SSIP) mainly appears in conventional ether electrolyte due to strong solvation ability of DME with Li^+^, resulting in facile dissolution and shuttling of LiPSs, thereby severely corrode Li metal anode and form a poor solvent‐derived SEI layer. This situation can be effectively inhibited in the WSE (Figure 1c), wherein a higher content of TFSI^−^ anions participates in the solvation structure to form contact ion pairs (CIPs) and Aggregates (AGGs) because of the weakly solvating ability of CPME with Li^+^, which endows anions‐derived SEI layer and uniform Li deposition morphology on Li metal anode. Meanwhile, due to the moderate LiPSs solubility in CPME solvent, the WSE simultaneously exhibits partly inhibited shuttle effect of LiPSs and appropriate conversion kinetics. Moreover, we also find that DOL additive co‐decomposes with anions to form a thin organic‐inorganic hybrid cathode electrolyte interphase (CEI) on the S cathode surface, which could effectively facilitate the conversion kinetics of S and suppress dead S/Li_2_S growth in WSE. The S/C||Li battery using the 1 M LiTFSI‐CPME with 2wt% DOL delivers a stable cycling performance with a high average CE ≈98.6% and a high capacity above 580 mAh g^−1^ (capacity retention rate ≈82.4%) after 200 cycles at 0.5C. Particularly, even under practical harsh conditions of high S loading (5 mg cm^−2^), thin Li metal anode (50 µm) and low electrolyte‐to‐S (E/S) ratio (5 µL mg^−1^), the S/C||Li pouch cell can also deliver impressive cycling stability.

## 2 Results and Discussion

### 2.1 Solvation Structures Of Different Electrolytes With LiPSs

**Figure 2:**
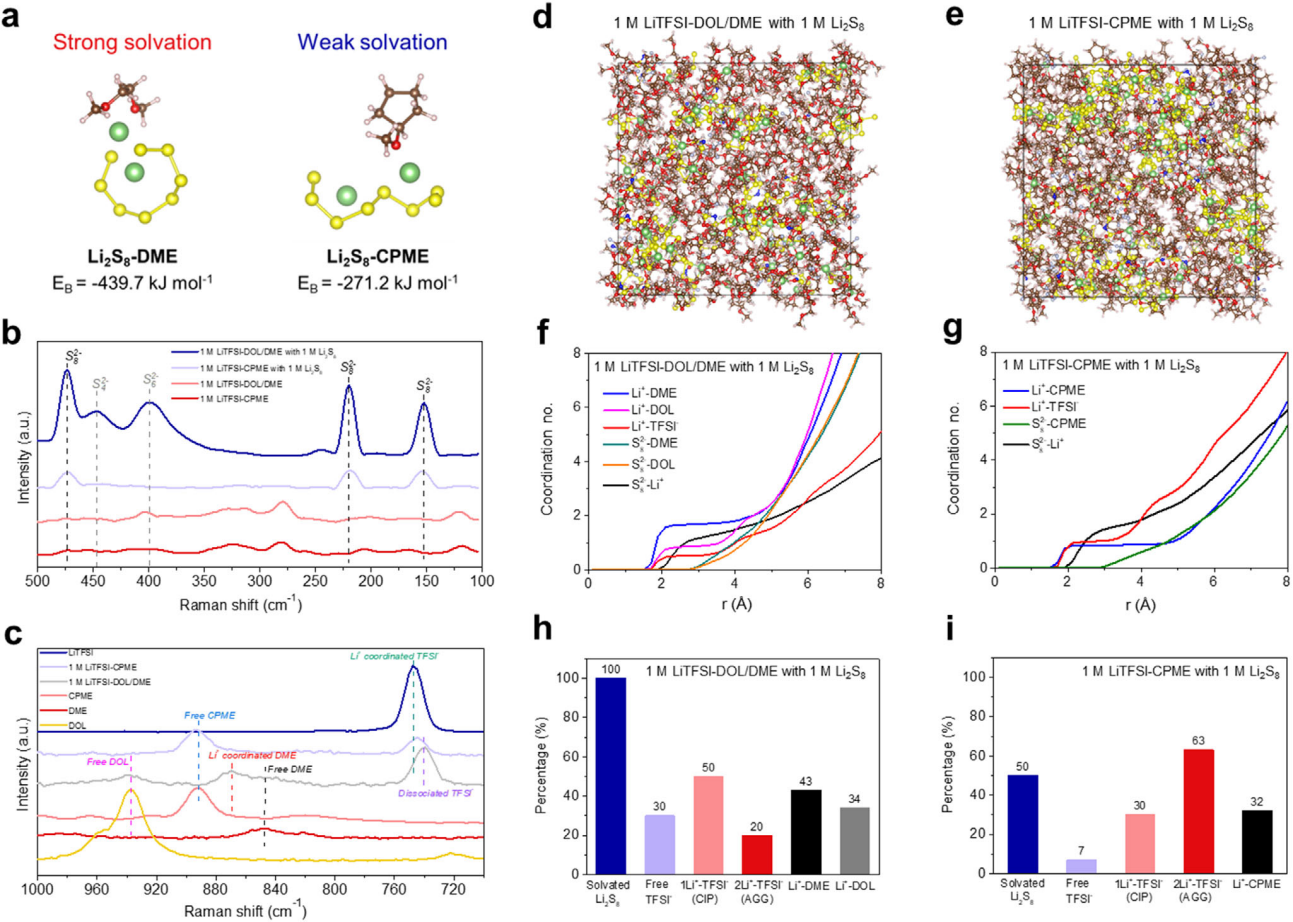
Analysis of electrolyte solvation structure. a) The binding energy of Li_2_S_8_ with DME and CPME obtained by DFT calculations. b) Raman spectra of different electrolytes before and after adding 1 M Li_2_S_8_. c) Raman spectra of different components and electrolytes. Snapshots, coordination numbers and coordination percentages obtained by MD simulations of d,f,h) 1 M LiTFSI‐DOL/DME with 1 M Li_2_S_8_ and e,g,i) 1 M LiTFSI‐CPME with 1 M Li_2_S_8_. Color code: Li‐green, O‐red, N‐dark blue, S‐yellow, F‐cyan, C‐brown, and H‐light pink.

The conversion kinetics of S cathode is highly depended on the solubility of LiPSs in the electrolytes. The binding energy (E_B_) between Li_2_S_8_ and different solvents was first studied by density functional theory (DFT) calculations, shown in **Figure** **2**a. The E_B_ between Li_2_S_8_ and DME is −439.7 kJ mol^−1^ and is much higher than the value of E_B_ between Li_2_S_8_ and CPME (−271.2 kJ mol^−1^), which is due to the high electron‐donating ability and the chelation effect of DME molecules, resulting in a high LiPSs solubility in DME‐based conventional electrolyte. The LiPSs solubility in different electrolytes was investigated by adding S and Li_2_S to prepare 1 M Li_2_S_8_ and continuously stirring for 72 h. Figure S1 (Supporting Information) reveals that the color of 1 M LiTFSI‐DOL/DME turns dark brown quickly after adding S and Li_2_S, indicating a large amount of Li_2_S_8_ is immediately dissolved in the electrolyte due to the high binding energy between DME and LiPSs. As expected, the color of 1 M LiTFSI‐CPME slowly becomes light brown with S and Li_2_S after 72 h stirring, indicating the LiPSs solubility is greatly decreased in CPME‐based electrolyte because of the weak binding energy between CPME and LiPSs. This point is also supported by UV–vis) spectrum, showing a lower content of LiPSs is detected in the 1 M LiTFSI‐CPME than that in 1 M LiTFSI‐DOL/DME (Figure S2, Supporting Information).^[^ ^38^ ^]^ Raman spectra of different electrolytes also demonstrate this point, shown in **Figure** **2**b. Compare to the Raman spectra of 1 M LiTFSI‐DOL/DME and 1 M LiTFSI‐CPME electrolytes, new Raman peaks corresponding to LiPSs appear in the 100–500 cm^−1^ after adding S and Li_2_S, implying S can react with Li_2_S to generate LiPSs in the two electrolytes.^[^ ^39^ ^]^ Notably, the intensity of Raman peaks ascribed to the LiPSs in 1 M LiTFSI‐CPME are significantly lower than that in 1 M LiTFSI‐DOL/DME, demonstrating LiPSs solubility is greatly reduces in 1 M LiTFSI‐CPME.

The solvation structure of different electrolytes was also characterized by Raman spectra. Blue‐shift or red‐shift range of typical Raman peaks of solvents or anions before and after adding lithium salt can effectively reflect the coordination strength of these components with Li^+^.^[^ ^27^, ^28^ ^]^ As shown in Figure 2c, the C─O stretching vibration peak of CPME slightly blue‐shifts from 891.9 to 893.5 cm^−1^ with a shift value of 1.6 cm^−1^ in 1 M LiTFSI‐CPME, but the C‐O peak of DME greatly blue‐shifts from 847.3 to 869.4 cm^−1^ with a much higher value of 22.1 cm^−1^ in 1 M LiTFSI‐DOL/DME. Meanwhile, the S─N stretching vibration peak of TFSI^−^ red‐shifts from 747.1 to 740.5 cm^−1^ with a shift value of 6.6 cm^−1^ in 1 M LiTFSI‐DOL/DME, but the S‐N peak of TFSI^−^ red‐shifts from 747.1 to 745.3 cm^−1^ with a much lower value of 1.8 cm^−1^ in 1 M LiTFSI‐CPME. Above these results indicate that more TFSI^−^ anions and less solvents participate in the Li^+^ solvation structure in 1 M LiTFSI‐CPME, but less TFSI^−^ anions and more solvents participate in the Li^+^ solvation structure in 1 M LiTFSI‐DOL/DME, which is owing to the weakly solvating ability of CPME solvent.

The solvation structures of different electrolytes were further studied by molecular dynamics (MD) simulations. The MD simulation systems are respectively composed of 1 M LiTFSI‐DOL/DME with 1 M Li_2_S_8_ (Figure 2d) and 1 M LiTFSI‐CPME with 1 M Li_2_S_8_ (Figure 2e). The coordination numbers of solvation center of Li^+^ in the two electrolytes show distinct plateaus at the position ≈2.0 Å, which is defined as the primary Li^+^ solvation shell. In 1 M LiTFSI‐DOL/DME electrolyte with 1 M Li_2_S_8_, the order of coordination numbers of solvents and anions in primary Li^+^ solvation shell is DME > DOL > TFSI^−^, indicating a solvents‐dominated Li^+^ solvation structure in this electrolyte (Figure 2f). In contrast, an anions‐dominated Li^+^ solvation structure is obtained in 1 M LiTFSI‐CPME electrolyte with 1 M Li_2_S_8_, in which the coordination number of TFSI^−^ is slightly higher than that of CPME due to the weakly solvating ability of CPME solvent (Figure 2g). At the same time, the solvation shell of S_8_^2−^ anions in the two electrolytes was also analyzed. Notably, the primary solvation shell of S_8_^2−^ anion is occupied by Li^+^ at the position from 2.0 to 2.7 Å in both two electrolytes. The secondary solvation shell is attributed by the solvents at the position from 2.7 Å to 4.0 Å. Apparently, higher coordination number of S_8_^2−^ with solvent is obtained in the 1 M LiTFSI‐DOL/DME with 1 M Li_2_S_8_ than that in the 1 M LiTFSI‐CPME with 1 M Li_2_S_8_, and the coordination numbers of S_8_^2−^ with Li^+^ show the opposite pattern in these two electrolytes, implying more Li_2_S_8_ are dissociated and solvated by solvents in the DME‐based electrolyte than that in the CPME‐based electrolyte. Above results are consistent with the binding energy results in Figure 2a, suggesting the dissociation and solvation of Li_2_S_8_ are relatively suppressed in CPME solvent than that in DME solvent.

The MD simulation results were statistically analyzed, and the percentages of different coordination components were given in Figure 2h,i. In 1 M LiTFSI‐DOL/DME with 1 M Li_2_S_8_, the percentage of solvated Li_2_S_8_ (Li_2_S_8_ coordinated with solvents or anions) is 100%, which is much higher than that of 50% in 1 M LiTFSI‐CPME with 1 M Li_2_S_8_, proving that the solubility and solvation of Li_2_S_8_ are effectively suppressed in the CPME‐based WSE. Furthermore, the percentage of free TFSI^−^ is 30% and the percentage of contact ion pairs (CIPs, 1 Li^+^ coordinated with TFSI^−^) is much higher than the aggregates (AGGs, 2 Li^+^ coordinated with TFSI^−^) in 1 M LiTFSI‐DOL/DME with 1 M Li_2_S_8_, (Figure 2h). In contrast, the percentage of free TFSI^−^ is only 7% in 1 M LiTFSI‐CPME with 1 M Li_2_S_8_, and AGGs structure displays a much higher percentage than the CIPs (Figure 2i). At the same time, the percentage of Li^+^‐CPME is relatively lower than that of Li^+^‐DME and Li^+^‐DOL. All above results demonstrate that CIPs and DME/DOL solvated Li_2_S_8_ structures are typically existed in 1 M LiTFSI‐DOL/DME with 1 M Li_2_S_8_, while more anions participate into the Li^+^ solvation structures to form AGGs and CPME/anions solvated Li_2_S_8_ structures in the CPME‐based WSE than that in DME/DOL‐based electrolyte, which is owing to the weakly solvating ability of CPME solvent.

### 2.2 Electrochemical Performance of Li‐S Batteries

**Figure 3:**
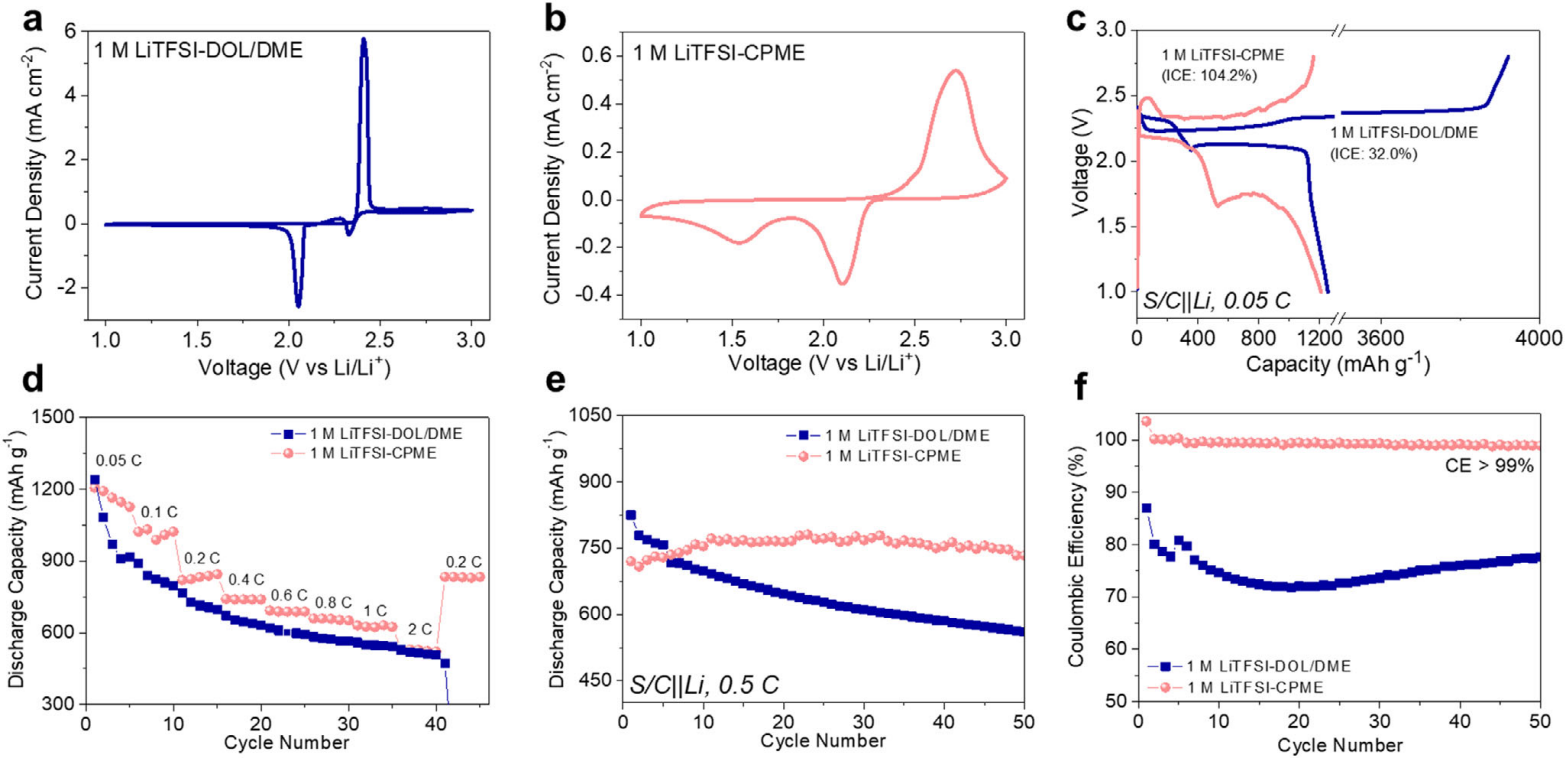
Electrochemical performance of Li‐S batteries with different electrolytes. CV curves of S/C||Li cells with a) 1 M LiTFSI‐DOL/DME and b) 1 M LiTFSI‐CPME. c) Initial charge‐discharge curves of S/C||Li cells with different electrolytes at 0.05 C. d) Rate capability of S/C||Li cells with different electrolytes. e) Discharge capacity and f) corresponding CE of S/C||Li cells with different electrolytes when cycling at 0.5 C.

To investigate the applicability of 1 M LiTFSI‐CPME electrolyte in Li‐S batteries, the S/C||Li cell with 1 M LiTFSI‐CPME was assembled, at the same time the S/C||Li cell with 1 M LiTFSI‐DOL/DME was as comparison. Cyclic voltammetry (CV) profiles were collected in a voltage range of 1.0–3.0 V at a scan rate of 0.1 mV s^−1^. Two reduction peaks and one oxidation peak are clearly observed in 1 M LiTFSI‐DOL/DME (**Figure** **3**a) and 1 M LiTFSI‐CPME (Figure 3b), corresponding to two transformation reactions of S to long‐chain LiPSs (Li_2_S_x_, 6 ≤ x ≤ 8) and long‐chain LiPSs to short‐chain LiPSs (Li_2_S_x_, 1 ≤ x ≤ 4) and the corresponding inverse process. Apparently, the distance between the reduction peaks and the oxidation peak is much farther in 1 M LiTFSI‐CPME than that in 1 M LiTFSI‐DOL/DME, indicating increased voltage polarization or changed LiPSs conversion path occur in 1 M LiTFSI‐CPME electrolyte. The similar results can be seen in the first charge/discharge plots of the S/C||Li cell with two electrolytes, shown in Figure 3c. The cell with 1 M LiTFSI‐CPME displays two discharge voltage plateaus (at ≈2.17 and ≈1.75 V) with much lower potential than that with 1 M LiTFSI‐DOL/DME (at ≈2.32 and ≈2.13 V). This phenomenon is ascribed to the weakly solvating ability of CPME resulting in lower ionic conductivity and lower LiPSs solubility in 1 M LiTFSI‐CPME than that in 1 M LiTFSI‐DOL/DME (Table S1, Supporting Information).^[^ ^34^, ^37^ ^]^ Interestingly, a high initial discharge capacity of 1208 mAh g^−1^ is still obtained in the S/C||Li cell with 1 M LiTFSI‐CPME at 0.05 C (Figure 3c), which indicates that an acceptable conversion kinetics of sulfur cathode is achieved in this electrolyte. Meanwhile, the S/C||Li cell with 1 M LiTFSI‐DOL/DME delivers a significantly high charge capacity above 3900 mAh g^−1^, which causes a low initial CE (ICE) of 32.0% (discharge capacity/charge capacity) of the battery. The above phenomenon is mainly due to the severe shuttle effect, which causes continuous side reactions between polysulfides and the Li metal anode. At the same time, the unstable solvent components in the electrolyte also undergo additional side reactions with the active Li metal electrode, thus resulting in the battery's charging capacity being higher than the theoretical capacity. In contrast, the ICE of the S/C||Li cell with 1 M LiTFSI‐CPME is ≈104.2%, implying the shuttle effect of LiPSs and side reactions are greatly suppressed. Notably, even if the discharge specific capacity of the battery using CPME‐based electrolyte is close compared to that using DOL/DME‐based electrolyte, the reduction in discharge voltage plateau significantly reduces the energy density of the battery (2182.6 Wh kg^−1^ vs 2518.7 Wh kg^−1^, calculated based on the mass of active sulfur). Therefore, future research still needs to further enhance the conversion kinetics of the cathode and reduce the polarization voltage through the design of the cathode structure (such as the use of catalysts) and the introduction of electrolyte additives, so as to better meet the requirements of battery energy density and electrochemical performance.

To further investigate the conversion mechanism of sulfur in 1 M LiTFSI‐CPME, the S/C||Li cell was first discharged at different capacities (stage1: 200 mAh g^−1^; stage2: 530 mAh g^−1^; stage3: 820 mAh g^−1^; stage4: 1208 mAh g^−1^) (Figure S3a, Supporting Information), and then disassembled to test the Raman spectra of S/C cathode at different stages (Figure S3b, Supporting Information). Typical Raman peaks ascribed to medium‐chain and long‐chain LiPSs (Li_2_S_n_, 4 ≤ n ≤ 8) are detected at stage1, which almost disappear at stage2 and then formed a little Li_2_S_4_ and Li_2_S_8_ at stage3, finally completely disappear at stage4.^[^ ^39^ ^]^ Above results show that in the process of transforming solid S into solid Li_2_S in CPME‐based electrolyte is a solid‐liquid‐solid conversion mechanism. Some medium‐chain and long‐chain LiPSs are also formed in the conversion process, which guarantees an acceptable cathodic conversion kinetics due to the mild solubility of CPME‐based electrolyte to LiPSs. However, compared with the conventional solid‐liquid‐solid conversion mechanism reported in the DOL/DME‐based electrolyte^[^ ^4^ ^]^ the solid‐liquid‐solid conversion path in the CPME‐based electrolyte is different. Compared with the S/C||Li cell using DOL/DME‐based electrolyte, the discharge curve of the S/C||Li cell using CPME‐based electrolyte has a longer first plateau and a shorter second plateau (Figure 3c). This implies that both long‐chain LiPSs and intermediate LiPSs (Li_2_S_n_, 4 ≤ n ≤ 8) exist simultaneously in the first plateau at ≈2.17 V, while the content of these LiPSs is relatively small in the second plateau at ≈1.75 V. Hence, the conversion process in the CPME‐based electrolyte endures a conversion from solid S to liquid LiPSs (Li_2_S_n_, 4 ≤ n ≤ 8) occurred at 2.17 V. Along with the voltage decreased, liquid LiPSs transform into solid short‐chain LiPSs (Li_2_S_n_, 1 ≤ n ≤ 2), while in the second plateau, it mainly changed from solid short‐chain LiPSs to Li_2_S.

Beneficial from the solid‐liquid‐solid conversion process, the S/C cathode obtained a high initial discharge capacity and good rate capability in 1 M LiTFSI‐CPME electrolyte. Figure 3d exhibits a high capacity of 530 mAh g^−1^ is still achieved even at a high charge‐discharge rate of 2 C for the S/C||Li cell with 1 M LiTFSI‐CPME. The reversibility and capacity at different charge‐discharge rates in 1 M LiTFSI‐CPME is relatively better than that in 1 M LiTFSI‐DOL/DME. Simultaneously, owing to 1 M LiTFSI‐CPME can suppress the shuttle effect of LiPSs, a stable cycling performance with almost constant discharge capacity (above 730 mAh g^−1^) and high CE (> 99%) over 50 cycles is obtained in 1 M LiTFSI‐CPME at 0.5 C (shown in Figure 3e,f; Figure S4a, Supporting Information), which is better than the cycling performance of the battery in 1 M LiTFSI‐DOL/DME with a rapid capacity decay and low CE (shown in Figure 3e,f; Figure S4b, Supporting Information). The better rate capability, more stable capacity and higher CE of batteries in CPME‐based electrolyte mainly arise from mild LiPSs solubility, inhibited shuttle effect of LiPSs and stable SEI layer formed on the Li metal surface.

### 2.3 Interfacial Chemistry on Li Metal Anode in Different Electrolytes

**Figure 4:**
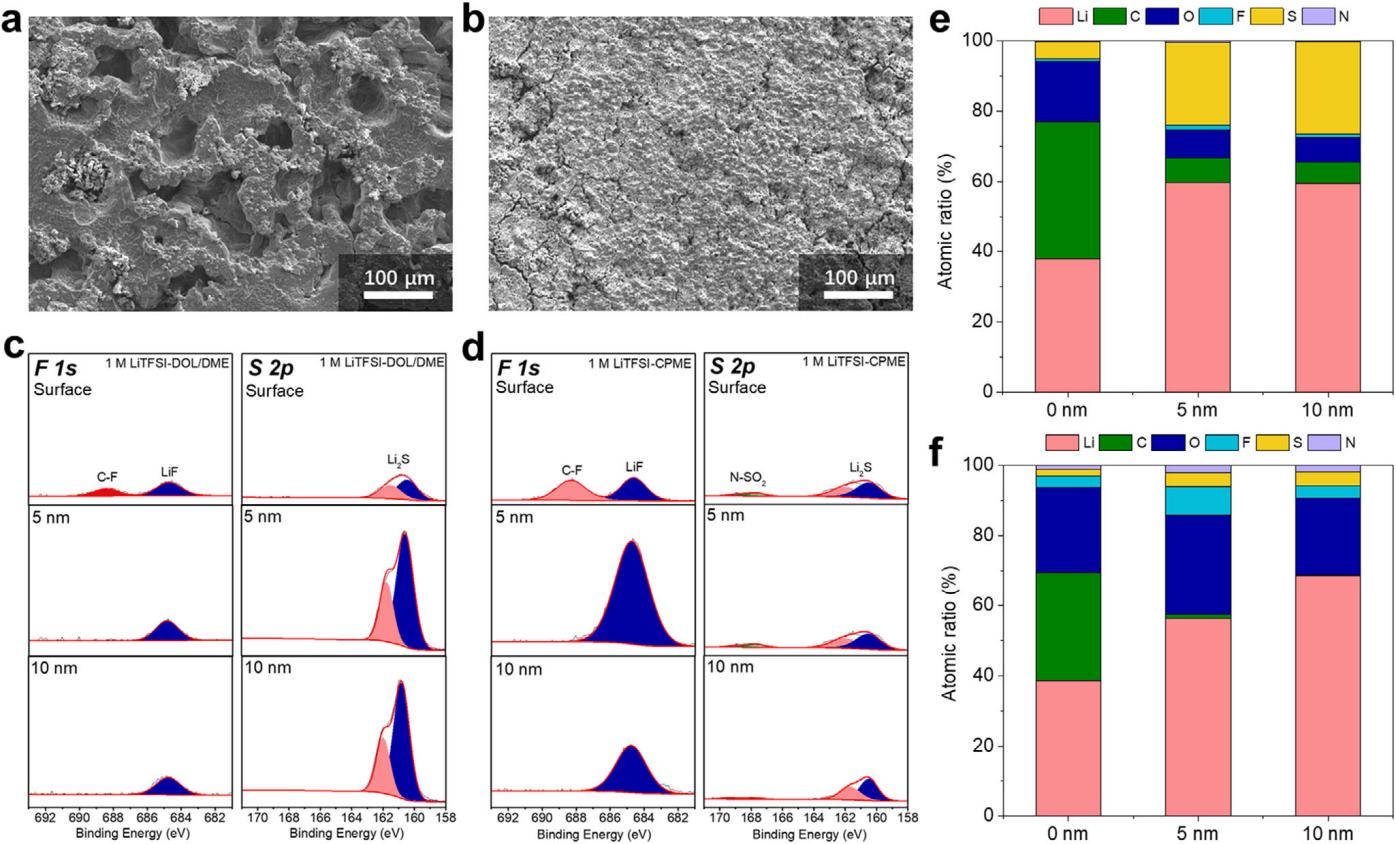
Interfacial chemistry of Li metal anode in different electrolytes. SEM of Li metal anode morphology after 50 cycles in S/C||Li cells with a) 1 M LiTFSI‐DOL/DME and b) 1 M LiTFSI‐CPME. XPS of Li metal anode at different sputtering depth after cycling in S/C||Li cells with c) 1 M LiTFSI‐DOL/DME and d) 1 M LiTFSI‐CPME. Atomic ratios of different elements obtained by XPS analysis at different sputtering depth of Li metal anode after cycling in S/C||Li cells with e) 1 M LiTFSI‐DOL/DME and f) 1 M LiTFSI‐CPME.

In order to investigate the interfacial stability between the electrolytes and Li metal anode in Li‐S batteries with continuous generation and consume of LiPSs, S/C||Li batteries were disassembled after 50 cycles, and the cycled Li metal anodes were collected for characterizations. First, the morphologies of Li metal anode after cycling in different electrolytes were observed by Scanning electron microscopy (SEM). The cycled Li metal anode in S/C||Li cell with 1 M LiTFSI‐DOL/DME is loose and porous (**Figure** **4**a). However, a compact and smooth morphology of Li metal anode is achieved in S/C||Li cell with 1 M LiTFSI‐CPME (Figure 4b). Furthermore, XPS analysis was performed to identify the chemical decomposition products on the Li metal anode after cycling in different electrolytes. Obviously, in addition to a bit of C‐F and LiF formed by TFSI^−^ anion decomposition, a large number of Li_2_S are enriched in the inner and outer layer of SEI film on the surface of Li metal anode after cycling in 1 M LiTFSI‐DOL/DME (Figure 4c). This indicates a severe corrosion of Li metal anode by LiPSs shuttling in the electrolyte, which leads to low CE of Li‐S batteries as well as loose and porous morphology of Li anode. However, in the SEI layer formed in the 1 M LiTFSI‐CPME, only a small amount of Li_2_S was detected both in the inner and outer layer of SEI, most of these components may be formed by the reduction products of TFSI^−^ anions (Figure 4d). Meanwhile, the atomic ratio of S also shows higher contents both in the outer and inner SEI layer on the Li metal anode after cycling in 1 M LiTFSI‐DOL/DME (Figure 4e) than that in 1 M LiTFSI‐CPME (Figure 4f). Therefore, these results demonstrate that the parasitic reactions between LiPSs and Li metal anode are significantly suppressed in S/C||Li cell with CPME‐based electrolyte, which may be due to the combination effect of moderate solubility of LiPSs and stable SEI layer formed on the Li metal surface.

To study the Li plating/stripping behavior and SEI chemistry on Li metal surface in different electrolytes without the interference of LiPSs, Li||Cu cells were assembled and tested at a current density of 0.5 mA cm^−2^ and a capacity density of 0.5 mAh cm^−2^. As shown in Figure S5 (Supporting Information), the Li||Cu cell with 1 M LiTFSI‐DOL/DME delivered a fluctuant Li plating/stripping CE with an average CE of ≈87%, indicating inevitable side reactions occur between this electrolyte and Li metal. Notably, a stable cycling performance of Li||Cu cell was obtained in 1 M LiTFSI‐CPME, with an apparently improved average CE of ∼98% and long cycle life above 400 cycles, demonstrating a high stability of Li metal with CPME‐based electrolyte. Then, X‐ray photoelectron spectroscopy (XPS) was performed to investigate the chemical compositions of SEI layers formed on the Li metal surface after 50 cycles in Li||Cu cell with different electrolytes. As shown in Figure S6 (Supporting Information), the SEI components show a double‐layer structure with different sputtering depth of XPS. The surface layer was dominated by C‐containing organic species derived by organic solvents, while the inner layer was dominated by inorganic elements such as O and F. By comparison, the atomic content of C derived from organic solvents in the SEI layer formed by 1 M LiTFSI‐DOL/DME is higher than that in the SEI layer formed by 1 M LiTFSI‐CPME, while the elements derived from TFSI^−^ anions (O, F, S, N) show higher contents in the SEI layer formed by 1 M LiTFSI‐CPME than that in the SEI layer formed by 1 M LiTFSI‐DOL/DME. Meanwhile, as shown in Figure S7 (Supporting Information), the decomposition products derived from TFSI^−^ anions (such as LiF, S═O, Li_2_S, etc.) show a higher content on the Li metal surface cycled in 1 M LiTFSI‐CPME than that cycled in 1 M LiTFSI‐DOL/DME, and the organic components (such as C─O, C─C/C─H, etc.) derived from solvents display the opposite pattern.

In order to understand the reduction characteristics of different components in these two electrolytes, the lowest unoccupied molecular orbital (LUMO) was calculated by DFT calculations (Figure S8, Supporting Information). It is found that the AGG complex (2Li^+^‐TFSI^−^) shows the lowest LUMO energy than other components in 1 M LiTFSI‐CPME, indicating it will be preferentially reduced to form an anions‐derived SEI layer on the electrode.^[^ ^36^, ^38^ ^]^ However, the Li^+^‐DOL complex exhibits the lowest LUMO energy in 1 M LiTFSI‐DOL/DME, thereby it contributes more organic components in the SEI. Above results are consistent with our prediction, confirming that the reduction product of anions is dominated to form an inorganic‐rich SEI layer on the Li metal surface in CPME‐based electrolyte, which is owing to the weakly solvating ability of CPME solvent results in plentiful AGGs to be preferential decomposed. The inorganic‐rich SEI layer can provide high mechanical strength and suppress interfacial side‐reaction, which is beneficial to induce homogeneous Li deposition behavior and suppress Li dendrite growth.^[^ ^14^, ^40^ ^]^ As a result, a denser and more uniform Li deposition morphology is obtained in Li||Cu cell with 1 M LiTFSI‐CPME than that with 1 M LiTFSI‐DOL/DME (Figure S9, Supporting Information).

### 2.4 Improvement of Cycling Performance of Li‐S Batteries in WSEs

**Figure 5:**
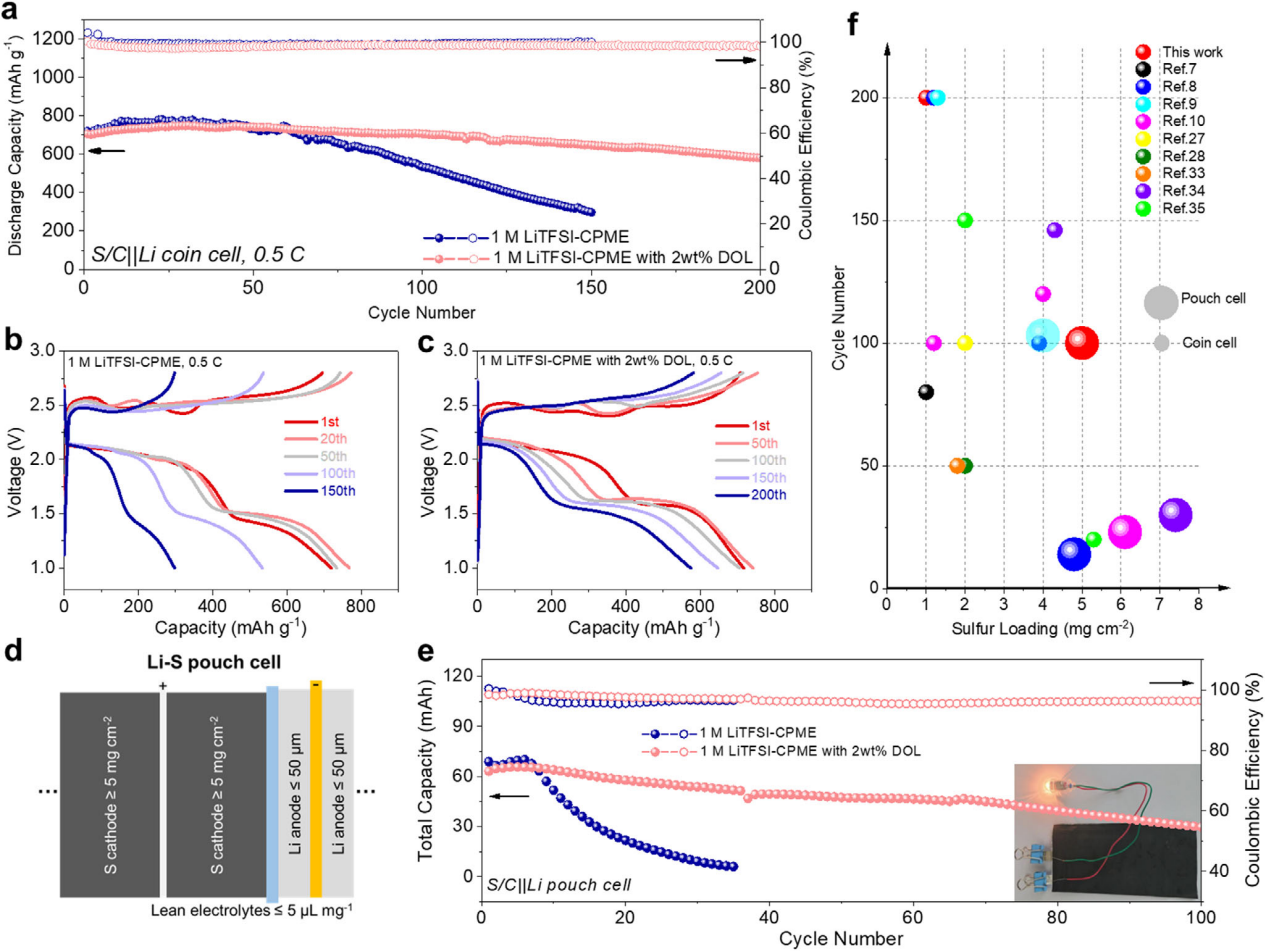
Electrochemical performance of Li‐S batteries in different electrolytes. a) Cycling performance of S/C||Li cells at 0.5C in different electrolytes. Charge‐discharge profiles of S/C||Li batteries at different cycles in b) 1 M LiTFSI‐CPME and c) 1 M LiTFSI‐CPME with 2wt% DOL. d) Structure diagram of the practical Li‐S pouch cell obtained in this work. e) Cycling performance of Li‐S pouch cells at 0.05C in different electrolytes. Inset is LED bulb lighted by Li‐S pouch cell. f) Comparisons of cycling performance with previous references using different electrolytes in Li‐S batteries.

Although the WSE can guarantee a good anodic stability of Li metal, the S/C||Li cell with this WSE still displayed continuous capacity decay and increased polarization after 60 cycles, and the capacity retention rate was only 40% after 150 cycles (**Figure** **5**a,b). Interestingly, when 2wt% of DOL additive was introduced in the WSE, the cycling stability of S/C||Li cell was significantly improved. The S/C||Li cell can deliver a most stable cycling performance, showing a high capacity above 580 mAh g^−1^ after 200 cycles at 0.5C (capacity retention rate of 82.4%, average CE of 98.6%) (Figure 5a,c). However, as shown in Figure S10 (Supporting Information), when the content of DOL additive increases to 5wt%, the CE of the Li‐S battery decreases and the capacity attenuation accelerates, indicating that excessive DOL may intensify the dissolution and shuttle effect of polysulfides, which is not conducive to the battery performance. When the addition amount of DOL is too little, such as 0.5wt%, the cycle stability is also worse than that of 2wt% of DOL additive. Additionally, the conversion mechanism of sulfur in 1 M LiTFSI‐CPME with 2wt% DOL was also investigated by Raman spectra, as shown in Figure S11 (Supporting Information), demonstrating a solid‐liquid‐solid conversion path similar to that of 1 M LiTFSI‐CPME (Figure S3, Supporting Information).

To investigate the practical application potential of WSEs in Li‐S batteries, S/C||Li pouch cells were assembled under harsh conditions (Figure 5d, high S cathode loading of ≈5 mg cm^−2^, 50 µm Li metal anode, and low E/S ratio of ≈5 µL mg_S_^−1^). As shown in Figure 5e, the Li‐S pouch cell in 1 M LiTFSI‐CPME only exhibited stable discharge capacity in initial 7 cycles, then it also displayed the same rapid capacity decay as the coin cell. As expected, although under these harsh conditions, the Li‐S pouch cell in 1 M LiTFSI‐CPME with 2wt% DOL can still show a stable cycling performance with high discharge capacity and high CE above 100 cycles. Meanwhile, this practical Li‐S pouch cell can effectively light the LED bulb when it was fully charged (Figure 5e inset), manifesting the practical feasibility. All the above results demonstrate that a bit of DOL (2wt%) can effectively improve the long‐term cycling performance of Li‐S battery in WSEs, which promotes the application potential of WSEs in practical Li‐S batteries. The cycling performance of Li‐S batteries in this work is compared with recently reported literatures about electrolyte modifications (Figure 5f), which exhibits longer cycle number than other works both in Li‐S coin cells and pouch cells, indicating that the electrolyte designed in this work has certain advancements in improving the cycle life of Li‐S battery.

### 2.5 The Effect Mechanism of DOL Additive in WSEs for Li‐S Batteries

**Figure 6:**
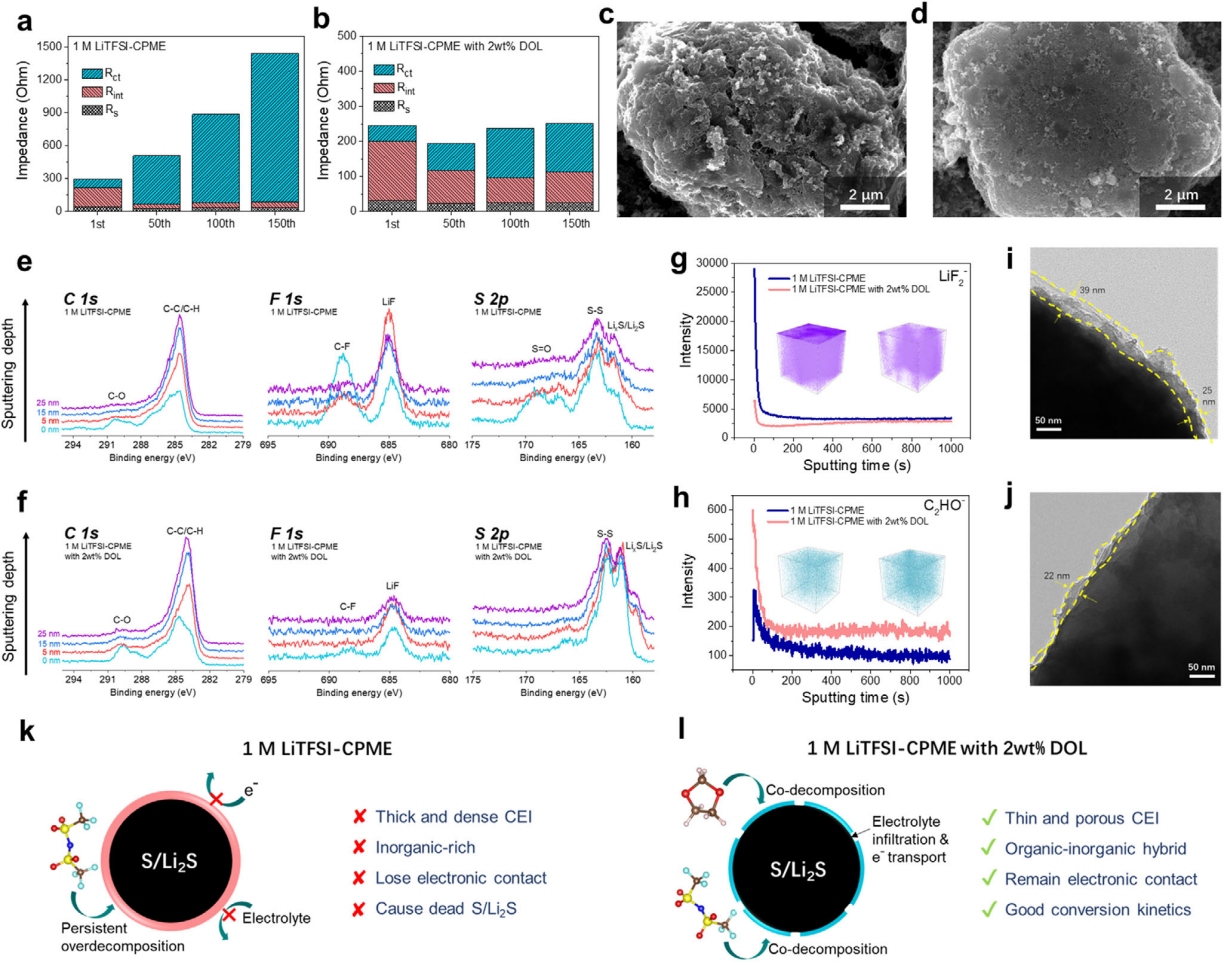
Characterizations of CEI layers formed on the S/C cathode after cycling. Impedances of S/C||Li batteries at different cycles in a) 1 M LiTFSI‐CPME and b) 1 M LiTFSI‐CPME with 2wt% DOL. Morphologies of S/C cathodes after 150 cycles in c) 1 M LiTFSI‐CPME and d) 1 M LiTFSI‐CPME with 2wt% DOL. XPS spectra of CEI layers on S/C cathode at different sputtering depth after 150 cycles in e) 1 M LiTFSI‐CPME and f) 1 M LiTFSI‐CPME with 2wt% DOL. TOF‐SIMS depth profiles and distributions (insets) of g) LiF_2_^−^ and h) C_2_HO^−^ species on the surface of S/C cathode after 150 cycles in 1 M LiTFSI‐CPME (left) and 1 M LiTFSI‐CPME with 2wt% DOL (right). TEM of the CEI morphologies on the surface of S cathode after 150 cycles in i) 1 M LiTFSI‐CPME and j) 1 M LiTFSI‐CPME with 2wt% DOL. Schematic diagram of CEI formation mechanism in k) 1 M LiTFSI‐CPME and l) 1 M LiTFSI‐CPME with 2wt% DOL.

For more in‐depth analysis of the failure mechanism of Li‐S batteries in WSEs and the effect mechanism of DOL additive, electrochemical impedance spectroscopy (EIS) was first performed to identify the impedances of Li‐S batteries in CPME‐based electrolyte without/with 2wt% DOL after different cycles. As shown in **Figures** **6**a,b, and S12 (Supporting Information), both in S/C||Li coin cells using 1 M LiTFSI‐CPME and 1 M LiTFSI‐CPME with 2wt% DOL, the bulk solution impedances (Rs) are almost unchanged at different cycles, it means that the electrolytes are always in a sufficient and stable state during the continuous cycles. At the same time, the interface impedances (R_int_) of these batteries decrease and tend to be stable with the extension of the number of cycles, indicating that stable SEI layers are formed on electrodes surface. However, the charge transfer impedances (R_ct_) of S/C||Li coin cells show significant differences between these two electrolytes. The R_ct_ of S/C||Li coin cell in 1 M LiTFSI‐CPME increases sharply as the number of cycles increases, but a slightly increased and low R_ct_ can be obtained after adding 2wt% DOL. Combined with the above EIS analysis results and the battery performance, it can be found that the rapid decline of the long‐term cycle capacity of the battery in the WSE may be related to the damage of the electronic conductive network of the electrode materials, and DOL can effectively inhibit this undesirable phenomenon. As far as we know, the interfacial reaction between the electrolyte and the active electrode materials in Li‐S batteries, will cause passivation on the surface of electrode materials to form an insulating layer. The dense insulating layer will cause the active material to lose electrical contact, particularly in active sulfur material due to its low electrical conductivity and dissolution/deposition conversion behavior, preventing it from participating in further redox reactions, ultimately leading to a sharp increase in charge transfer impedance and battery capacity attenuation. To deeply explore the fundamental causes of these phenomena, we then conducted a systematic study on the positive and negative electrode structures and their interface evolution behaviors, respectively.

In order to further analyze the state of the electrodes after cycling in WSE without/with 2wt% DOL, the S/C cathodes (fully charged to 3 V) and the Li metal anodes after 150 cycles in S/C||Li coin cells were collected, and the morphologies of the above electrodes were observed by SEM. At the S/C cathode side, a rough and heterogeneous morphology was observed on the S/C surface after cycling in 1 M LiTFSI‐CPME (Figure 6c), while a smooth and homogeneous morphology was seen in the S/C surface after cycling in 1 M LiTFSI‐CPME with 2wt% DOL (Figure 6d). This phenomenon indicates that plentiful decomposition products are formed and covered on the surface of the cycled S/C in 1 M LiTFSI‐CPME, and less decomposition products are formed after adding DOL additive. The chemical components of the decomposition products formed on the S/C cathodes were detected by Energy dispersive spectrometer (EDS) element mapping, XPS analysis and Time‐of‐flight secondary ion mass spectrometry (TOF‐SIMS). It can be observed that higher C atomic ratio and lower F atomic ratio distribute on the surface of S/C materials after cycling in 1 M LiTFSI‐CPME with 2wt% DOL than that without DOL (Figure S13, Supporting Information). Meanwhile, a large number of products decomposed by TFSI^−^ anions (e.g., LiF, S─F, C‐F, S═O, etc.) are formed on the surface of the S/C cathode after cycling in 1 M LiTFSI‐CPME (Figure 6e). This layer of products is thick and uniformly covered on the surface of the electrode, so that less S‐S and Li_x_S XPS signals were detected. In stark contrast, high intensity S‐S and Li_x_S XPS signals were detected on the surface of S/C cathode after cycling in 1 M LiTFSI‐CPME with 2wt% DOL, which also shows significant less anions decomposition products and more organic components than that in 1 M LiTFSI‐CPME (Figure 6f).

These results are also proved by using TOF‐SIMS, which is a highly surface‐sensitive technique with ultrahigh chemical selectivity. As shown in Figures 6g,h, lower content of LiF_2_^−^ and higher content of C_2_HO^−^ were detected on the S/C cathode after cycling in WSE with 2wt% DOL than that without DOL. The above results indicate that the addition of DOL can effectively reduce the decomposition of anions and form an organic‐inorganic hybrid CEI layer on the surface of S. The morphology and thickness of CEI layers formed on the surface of S were observed by transmission electron microscope (TEM). It is found that a dense and thick (25–39 nm) CEI layer is completely covered on the S surface after cycling in 1 M LiTFSI‐CPME (Figure 6i), but a thin (less than 22 nm) CEI layer is observed on the S surface after cycling in 1 M LiTFSI‐CPME with 2wt% DOL (Figure 6j). In order to further verify that the deterioration of Li‐S battery performance in 1 M LiTFSI‐CPME is related to the change of cathode side, the cycled S/C cathode was collected from the S/C||Li coin cell after 150 cycles to reassemble with a fresh Li metal anode and fresh electrolyte. From Figure S14 (Supporting Information), the reassembled S/C||Li cell still manifested low charge‐discharge capacity and large polarization, indicating that the electrochemical properties of the S/C cathode are seriously affected by the thick CEI layer formed on the surface.

At the Li metal anode side, SEM images show that the surface of the Li metal after cycling in 1 M LiTFSI‐CPME is covered with a compact and black layer (Figure S15a and inset, Supporting Information), which is ascribed to the anions‐derived SEI layer as reported by other literature.^[^ ^41^ ^]^ In contrast, a homogeneous and dense morphology is observed on the Li metal surface after cycling in 1 M LiTFSI‐CPME with 2wt% DOL, the optical image of the cycled Li metal displays a smooth and shiny surface morphology (Figure S15b and inset, Supporting Information). The XPS spectra in Figure S16 (Supporting Information) demonstrate that the inorganic products (such as Li_2_S, LiF, etc.) formed by LiPSs and anionic reduction are both detected on the Li metal surface after cycling in these two electrolytes, while a high content of SO_3_^2−^/S_2_O_3_^2−^ components derived by TFSI^−^ anions appears on the surface of the Li metal only after cycling in 1 M LiTFSI‐CPME.^[^ ^10^ ^]^ The above results indicate that the addition of DOL additive also affects the composition of the SEI on the Li anode surface, reducing the inorganic components and increasing the organic components. This is not conducive to blocking the parasitic reaction between LiPSs and the Li anode (consistent with a higher content of Li_2_S observed on the surface of Li metal in 1 M LiTFSI‐CPME with 2wt% DOL, shown in Figure S16b, Supporting Information), thereby leading to a decrease in the CE of the Li‐S battery (consistent with the results in Figure S10, Supporting Information). According to the above results and the electrochemical performance of Li‐S batteries, we believe that although the introduction of DOL additive will slightly intensify the interfacial side reactions at the Li metal anode, its influence is relatively small due to the low solubility of polysulfides in the CPME‐based electrolyte. In comparison, the role of DOL at the S/C cathode is more significant because the S particles require a continuous electronic conduction network to achieve its effective conversion and ensure the operation of the battery. Therefore, an optimized amount (2wt%) of DOL additive can well balance the kinetics of the cathode and the stability of the anode, thereby enabling the Li‐S battery to exhibit the best cycling performance.

Based on the above analysis, we propose the possible failure mechanism of Li‐S battery in WSE in Figure 6k. The anions‐dominated Li^+^ solvation structure in WSE could induce the preferential decomposition of TFSI^−^ anions, which generates LiF‐rich CEI layer on the cathode surface. As the cycle progresses, this inorganic‐rich CEI layer will continue to form until it is fully and densely coated on the surface of the S/Li_2_S particles, eventually causing S/Li_2_S to lose electrical contact and become dead S/Li_2_S on both cathode and anode surface. This may be one of the main reasons for the large R_ct_ and rapid capacity decay of the Li‐S battery after a certain number of cycles in WSE. However, when a small amount of DOL is introduced into WSE as an additive, DOL will preferentially decompose to form an organic‐inorganic hybrid CEI layer on the surface of S due to its high reactivity (Figure 6l).^[^ ^10^, ^40^ ^]^ This CEI layer can not only effectively prevent the continuous decomposition of anions, but also allows the electrolyte infiltration and adapts to the volume expansion of S/Li_2_S particles by virtue of its porous and flexible characteristics, so as to remain continuous electrical contact and good conversion kinetics of active materials. Therefore, the WSEs with DOL additive are very promising electrolytes for long‐term Li‐S batteries because it can remain a good balance between the anodic stability of Li metal and the cathodic conversion kinetics of S/C.

## 3 Conclusion

In summary, a high‐performance Li‐S battery necessitates good suppression of LiPSs shuttling, good anodic stability, and appropriate cathodic conversion kinetics, which are correlated with the Li^+^ solvation structure and LiPSs solubility in the electrolyte. This work adopts non‐fluorinated weakly solvating solvents to regulate the electrolyte solvation structure, and achieves a CPME‐based WSE with moderate LiPSs solubility, which helps to suppress the shuttle effect of LiPSs and induces an anions‐derived SEI layer on the Li metal surface. Additionally, utilizing a small amount of DOL as an additive in the WSE is proved to form a thin organic‐inorganic hybrid CEI layer on the S cathode, which helps to maintain good cathodic conversion kinetics and suppress dead S/Li_2_S growth. Consequently, the S/C||Li coin cell with the WSE can deliver appropriate rate capability and stable cycling performance at 0.5C, achieving a high average CE of 98.6% and a high capacity above 580 mAh g^−1^ (capacity retention rate of 82.4%) after 200 cycles. Furthermore, the S/C||Li pouch cell with the WSE also delivers stable capacity and high CE above 100 cycles even under harsh conditions. This work highlights the importance of balancing anodic stability and cathodic conversion kinetics for long‐life Li‐S batteries, proposes the adoption of the non‐fluorinated weakly solvating solvents to achieve these goals, and inspires the practical application of high‐performance Li‐S batteries.

## 4 Experimental Section

### Material Preparations

LiTFSI (DodoChem Co., Ltd.) was used after drying at 110 °C for 24 h in a vacuum. DME (Adamas, 99%+), DOL (Adamas, 99%+), CPME (Adamas, 98%+) were dried with molecular sieves before use. The S/C composite was prepared by infiltrating the sublimed S (98%, Aladdin) into the carbon nanotube (CNT, battery level, Shandong Dazhan Nanomaterial Co., Ltd.) via a simple melt‐diffusion method. CNT and pure S were mixed with a mass ratio of 1:3. The S/C cathode was prepared as follows: the S/C composite active material, conductive agent (including three materials: Super‐P (battery level, Termeco GMBH), CNT, and graphene (battery level, Knano Graphene Technology Co., Ltd.) were corresponding to a mass ratio of 6:2:2 in the conductive agent system), and LA133 binder (battery level, Shanghai Titan Scientific Co., Ltd.) were mixed at a mass ratio of 8:1:1 in N, N‐dimethylacetamide (DMAC, battery level, Adamas) and deionized water. Then, the slurry was spread on carbon‐coated aluminum foil and dried at 80 °C for 6 h. S/C electrodes were prepared with a S loading of ≈1.0 mg cm^−2^ for coin cells and ≈5.0 mg cm^−2^ for pouch cells.

### Characterizations

The mixture of S and Li_2_S with a molar ratio of 7:1 was added into electrolytes to identify the solubility of Li_2_S_8_. The solvation structures of different electrolytes and the electrolytes with Li_2_S_8_ (take the supernatant) were detected by Raman spectroscopy (Labram, HR) with 532 nm laser excitation. UV–vis spectroscopic analysis was performed with PERSEE TU‐1901 to measure LiPSs solubility in different electrolytes. The ionic conductivity of different electrolytes was collected at 25 °C with a Mettler Toledo S400 SevenExcellence instrument using an Inlab 741‐ISM conductive electrode. The viscosity was measured by using an NDJ‐9S viscometer (Lichen‐BX Co., Ltd.) at 25 °C. XPS analysis was performed by using an Escalab 250Xi spectrometer (VG, Altrincham, UK) with Al Kα radiation. TOF‐SIMS (TOF. SIMS5‐100) was performed by a 10 kV Bi^+^ ions beam and 1 kV Cs^+^ sputtering. Morphologies of Li metal anode and S/C cathode were observed by using SEM (Regulus 8230) after rinsing the samples in DME and drying them in an argon glovebox. Morphologies of CEI layers on S particles were observed by using TEM analysis (Tecnai G2 F20 S‐TWIN).

### Electrochemical Measurements

CV of the Li‐S batteries in different electrolytes was measured by a VPM300 analyzer (Biologic, France) at a scan rate of 0.1 mV s^−1^. Cu||Li and S/C||Li cells were assembled in CR2025 coin‐type cells using polypropylene membrane (PP, Celgard 2400) as the separator. All coin‐cells were assembled in an Ar‐filled glovebox with a controlled H_2_O and O_2_ level below 1 ppm. The electrochemical tests of the above cells were performed by the Neware battery test system (CT‐4000, Shenzhen Neware Technology Co., Ltd.). Cycling performance of Li‐S batteries were tested in a voltage range of 1.0 to 2.8 V at a current density of 0.5 C (1 C = 1675 mA g^−1^). Cells were tested at 25 °C in a thermostatic chamber. EIS of S/C||Li batteries was collected by a VPM300 analyzer in a frequency range from 7 MHz to 0.1 Hz.

### Theoretical Calculations

DFT calculations were performed using DMol3 module in Materials Studio software.^[^ ^42^, ^43^ ^]^ The geometry optimizations of different ions and molecules were carried out with the generalized‐gradient‐approximation (GGA)/Perdew‐Burke‐Ernzerhof (PBE) functional and double numeric polarization (DNP) basis set.^[^ ^44^ ^]^ The convergence tolerance was set to 1.0 × 10^−5^ Ha, 2.0 × 10^−3^ Ha Å^−1^, and 5.0 × 10^−3^ Å for energy, maximum force, and maximum displacement, respectively. The binding energy (E_B_) of Li_2_S_8_ (LiPS) with different solvents was calculated by the following formula: E_B_ = E_total_ – E_LiPS_ − E_solvent_, E_total_, E_LiPS_, and E_solvent_ are the energy of the Li_2_S_8_‐solvent, single Li_2_S_8_, and single solvent, respectively.^[^ ^45^ ^]^ MD simulations were performed by using the Forcite package in Materials Studio.^[^ ^46^ ^]^ The amorphous cells with 34.3 × 34.3 × 34.3 Å^3^ and 37.6 × 37.6 × 37.6 Å^3^ linear dimensions were adopted for MD simulations, corresponding to the LiTFSI/Li_2_S_8_/DOL/DME with a molar ratio of 20:20:140:100 for 1 M Li_2_S_8_ in 1 M LiTFSI‐DOL/DME, LiTFSI/Li_2_S_8_/CPME with a molar ratio of 30:30:210 for the 1 M Li_2_S_8_ in 1 M LiTFSI‐CPME, respectively. The solution structures were optimized with the COMPASSIII force field and then equilibrated in NPT for 50 ps using the Nosé thermostat and a Parrinello‐Rahman barostat to maintain a temperature of 298.15 K and a pressure of 1 bar.^[^ ^47^, ^48^ ^]^ Afterward, production runs were performed in an NVT ensemble, and the temperature was controlled using a Nosé thermostat with a target temperature of 298.15 K. A time step of 1.0 fs (femtosecond) and total simulation time of 2 ns (nanosecond) were chosen.

## Conflict of Interest

The authors declare no conflict of interest.

## Supporting information



Supporting Information

## Data Availability

The data that support the findings of this study are available from the corresponding author upon reasonable request.
