# Supplementary material for: Balancing Anodic Stability and Cathodic Kinetics in Practical Lithium‐Sulfur Batteries With Non‐fluorinated Weakly Solvating Solvents
Source: Adv Sci (Weinh). 2025 Jul 2;12(37):e17305. doi: 10.1002/advs.202417305 (PMC12499459; doi:10.1002/advs.202417305)
Supplement: Supplementary file 1 — Supporting Information [file ADVS-12-e17305-s001.docx]

Supporting information for:

**Balancing Anodic Stability and Cathodic Kinetics in Practical Lithium-Sulfur Batteries with Non-fluorinated Weakly Solvating Solvents**

Zhicheng Wang^1,3,‡^, Shixiao Weng^2,‡^, Haiyang Zhang^2,7,‡^, Liping Wang^3,4,‡^, Xu Yao^3^, Haifeng Tu^2^, Dan Huang^2^, Suwan Lu^2^, Lingwang Liu^2^, Jiangyan Xue^2^, Fengrui Zhang^1,3^, Guan Wu^7^, Jieyun Zheng^1,3^, Qing Wang^6^, Liquan Chen^1,3^, Jingjing Xu^2,5,*^, Hong Li^1,3,*^, and Xiaodong Wu^2,3,*^

^1^ Beijing Advanced Innovation Center for Materials Genome Engineering Key Laboratory for Renewable Energy, Beijing Key Laboratory for New Energy Materials and Devices, Institute of Physics, Chinese Academy of Sciences, Beijing 100190, China

^2^ *i*-lab, Suzhou Institute of Nano-Tech and Nano-Bionics (SINANO), Chinese Academy of Sciences, Suzhou 215123, China

^3^ Tianmu Lake Institute of Advanced Energy Storage Technologies Co., Ltd., Liyang 213300, China

^4^ School of Materials and Energy, University of Electronic Science and Technology of China, Chengdu 611731, China

^5^ College of Material Science and Engineering, Hohai University, Changzhou 213000, China

^6^ Department of Materials Science and Engineering, National University of Singapore, Singapore 117576, Singapore

^7^ National Engineering Lab for Textile Fiber Materials & Processing Technology, Zhejiang Sci-Tech University, Hangzhou 310018, China

^‡^ These authors contributed equally

^*^ Correspondence to: J.X., jjxu2011@sinano.ac.cn, H.L., hli@iphy.ac.cn, X.W., xdwu2011@sinano.ac.cn


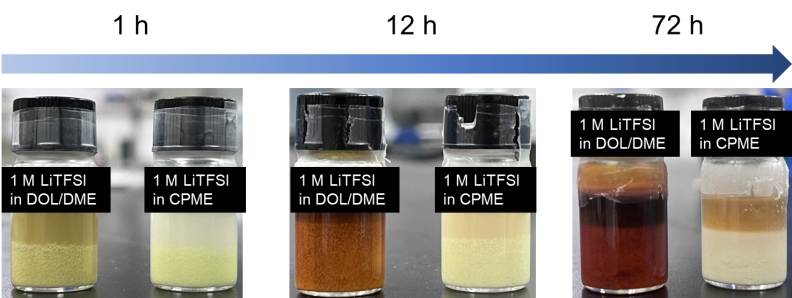


**Figure S1.** LiPSs solubility tests of 1 M LiTFSI-DOL/DME and 1 M LiTFSI-CPME electrolytes after adding S and Li_2_S to prepare 1 M Li_2_S_8_ and stirring for 1 h, 12 h and 72 h.


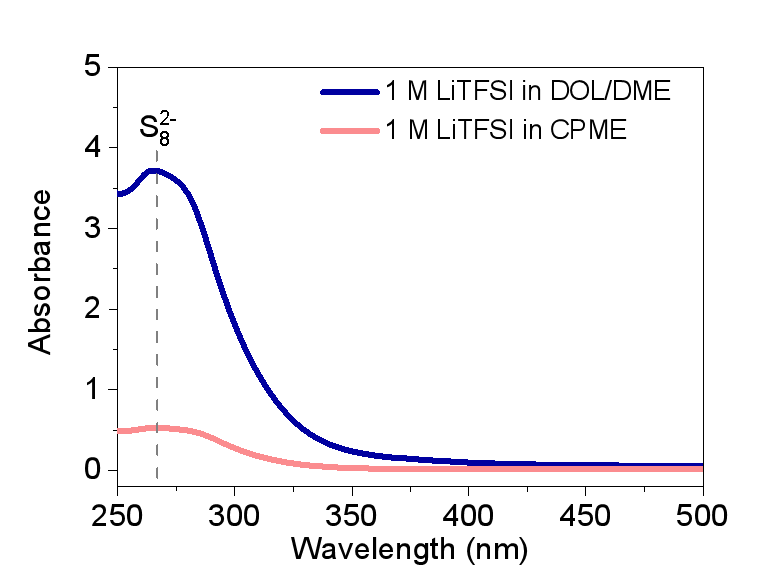


**Figure S2.** UV-vis spectrum of 1 M LiTFSI-DOL/DME and 1 M LiTFSI-CPME with S and Li_2_S after stirring for 72 h.

**Table S1.** Physicochemical properties of different electrolytes at 25 ℃


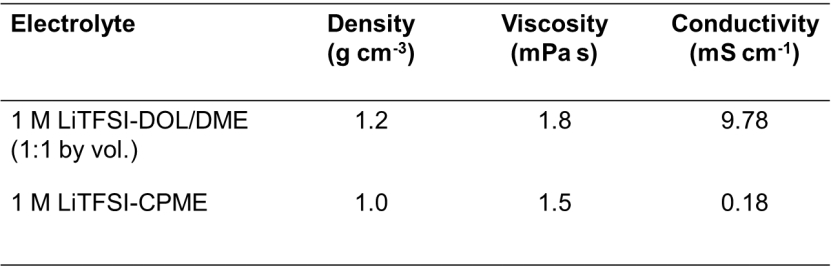


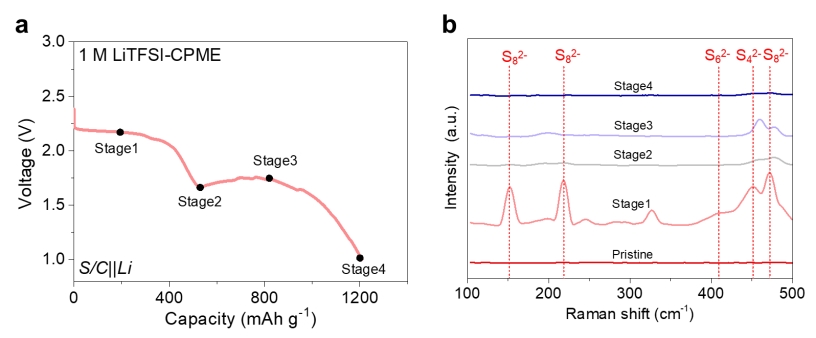


**Figure S3.** (a) Different stages at initial discharge profile of a S/C||Li cell in 1 M LiTFSI-CPME. (b) Raman spectra of S/C cathode at different stages.


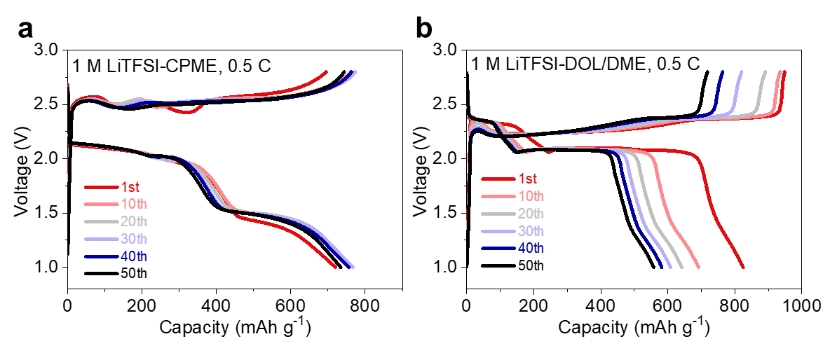


**Figure S4.** Charge-discharge curves of S/C||Li battery at different cycles and 0.5 C in (a) 1 M LiTFSI-CPME and (b) 1 M LiTFSI-DOL/DME.


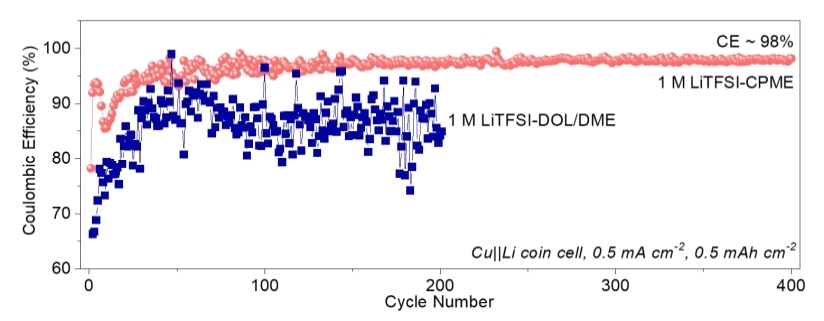


**Figure S5**. CE of Cu||Li cells with 1 M LiTFSI-DOL/DME and 1 M LiTFSI-CPME electrolytes.


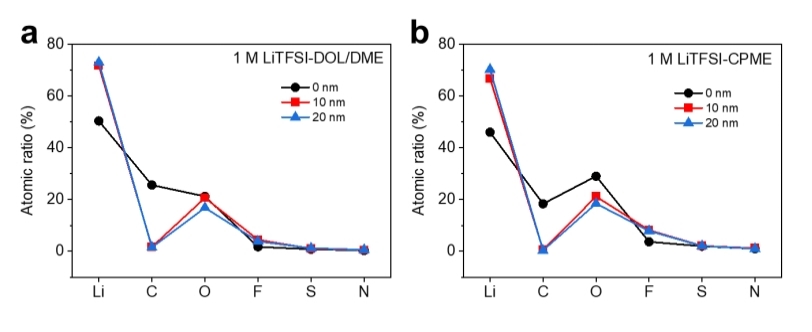


**Figure S6**. Atomic ratios of different elements obtained by XPS analysis at different sputtering depths in SEI layer formed on the Li metal surface after cycling in a) 1 M LiTFSI-DOL/DME and b) 1 M LiTFSI-CPME.


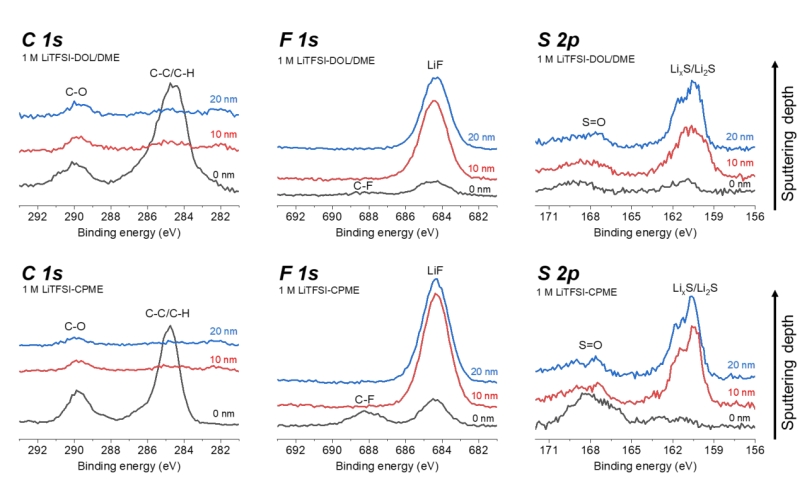


**Figure S7**. C 1s, F 1s, and S 2p XPS spectra at different sputtering depths in SEI layer formed on the Li metal surface after cycling in 1 M LiTFSI-DOL/DME and 1 M LiTFSI-CPME.


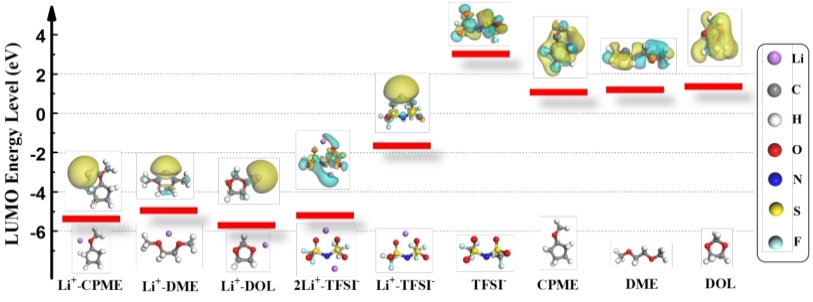


**Figure S8.** LUMO energies of different components in the electrolytes.


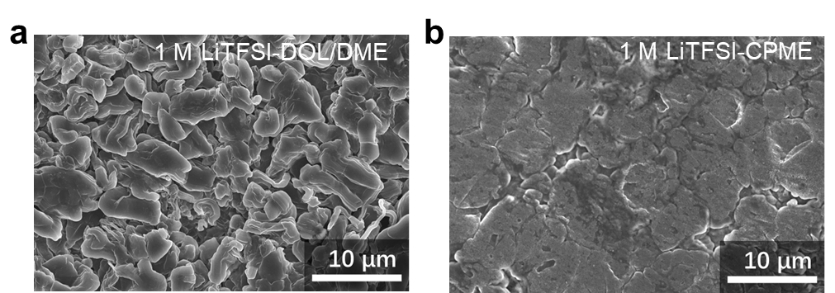


**Figure S9.** Morphologies of Li metal deposition on Cu foil in (a) 1 M LiTFSI-DOL/DME and (b) 1 M LiTFSI-CPME.


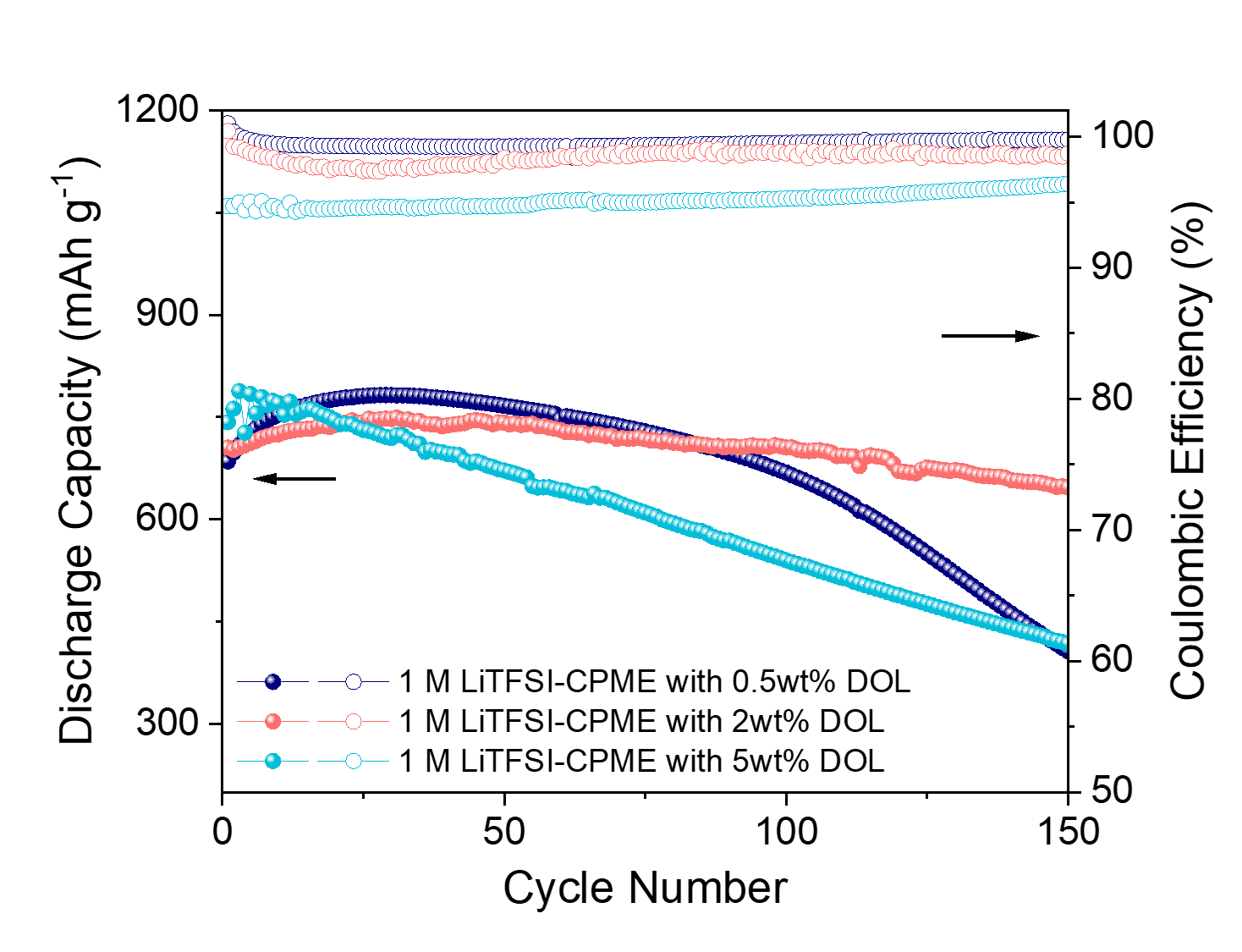


**Figure S10.** Cycling performance of S/C||Li cells in 1 M LiTFSI-CPME with different ratios of DOL additive at 0.5C.


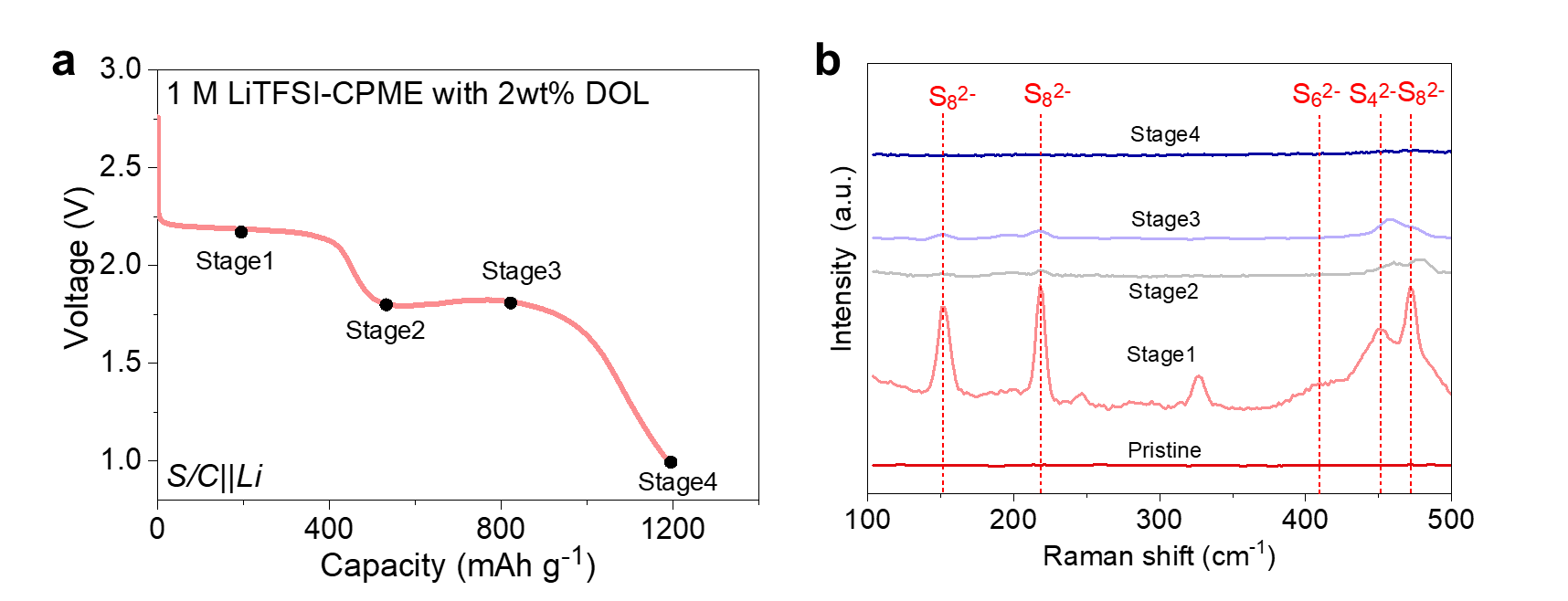


**Figure S11.** (a) Different stages at initial discharge profile of a S/C||Li cell in 1 M LiTFSI-CPME with 2wt% DOL. (b) Raman spectra of S/C cathode at different stages.


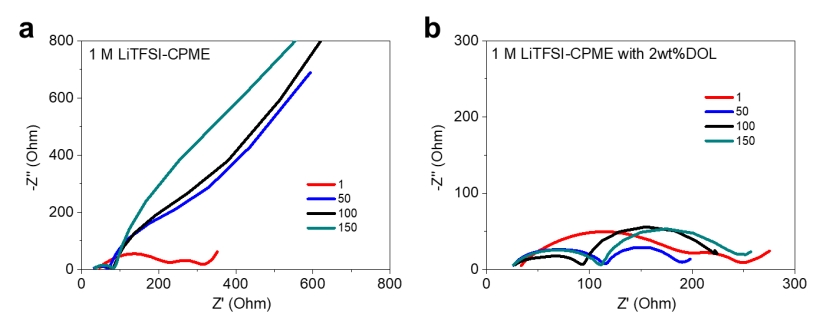


**Figure S12.** EIS curves of S/C||Li coin cells after different cycles in (a) 1 M LiTFSI-CPME and (b) 1 M LiTFSI-CPME with 2wt% DOL.


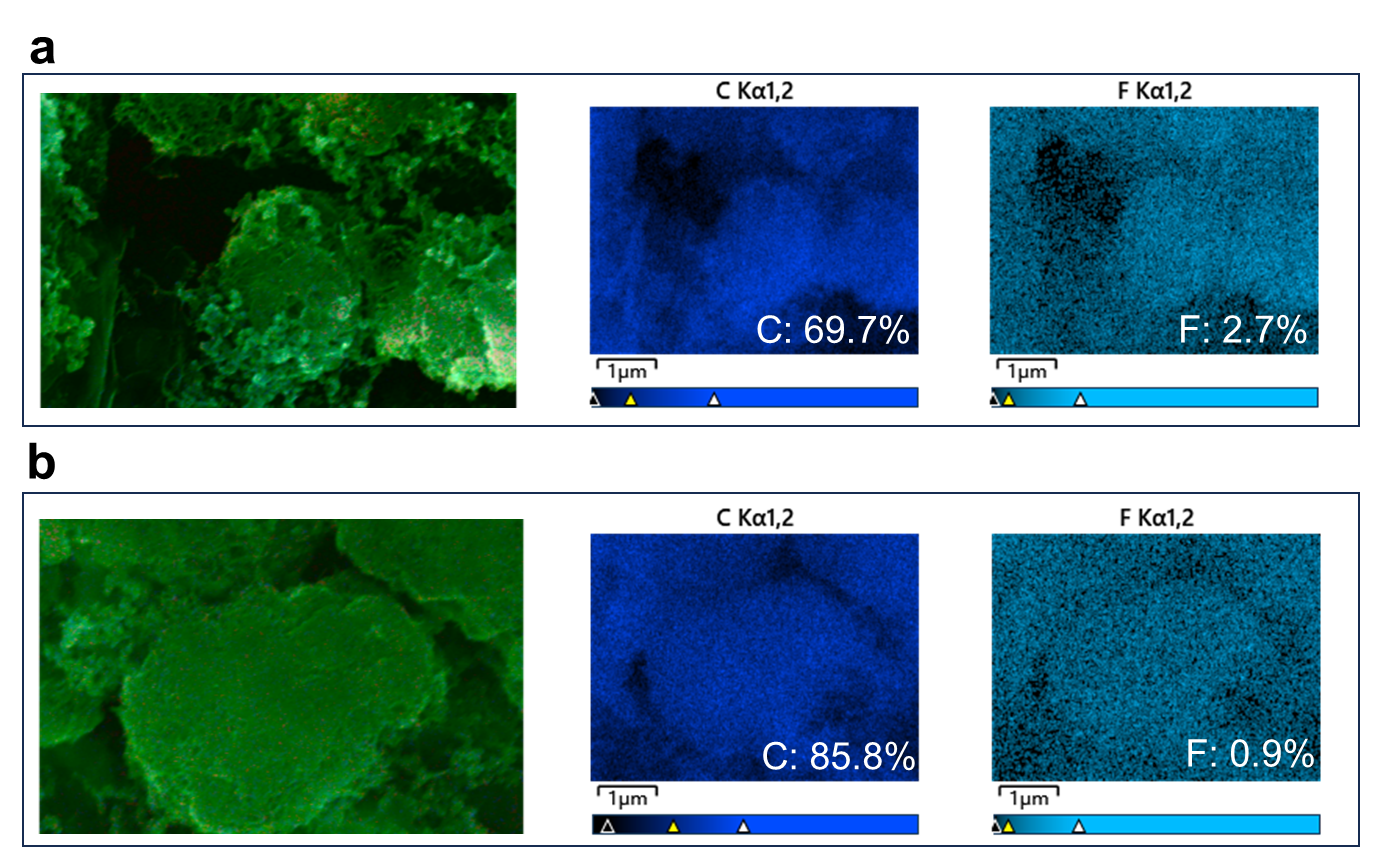


**Figure S13.** EDS images and C, F elemental mapping of S/C cathodes after 150 cycles in (a) 1 M LiTFSI-CPME and (b) 1 M LiTFSI-CPME with 2wt% DOL.


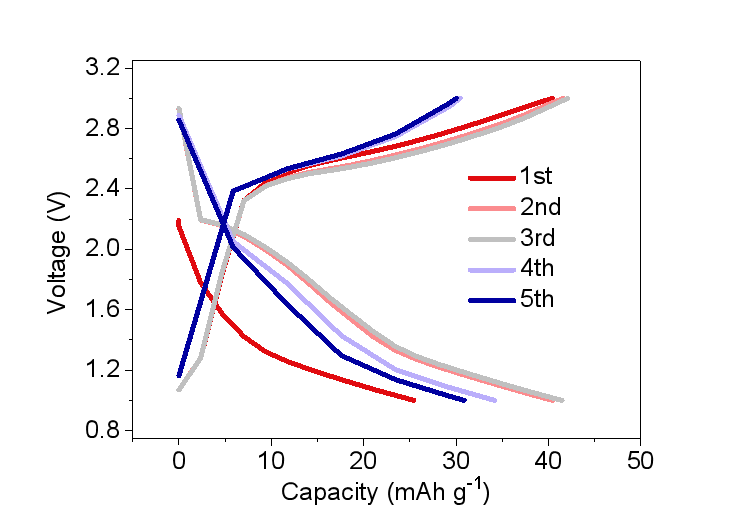


**Figure S14.** Charge-discharge curves of S/C||Li battery with cycled S/C cathode and fresh Li metal anode in 1 M LiTFSI-CPME.


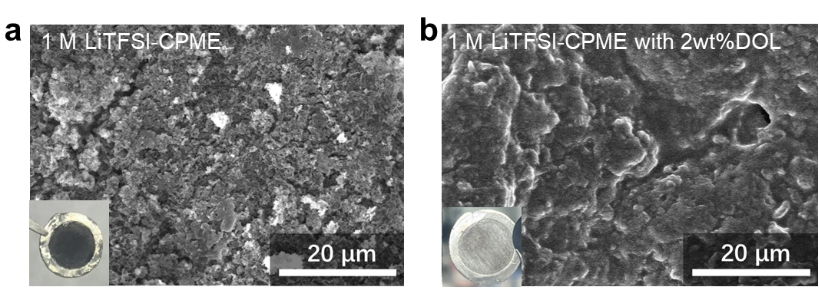


**Figure S15**. SEM of Li metal anode surface after 150 cycles in S/C||Li batteries in a) 1 M LiTFSI-CPME and b) 1 M LiTFSI-CPME with 2wt% DOL. Insets are optical images of cycled Li metal electrodes.


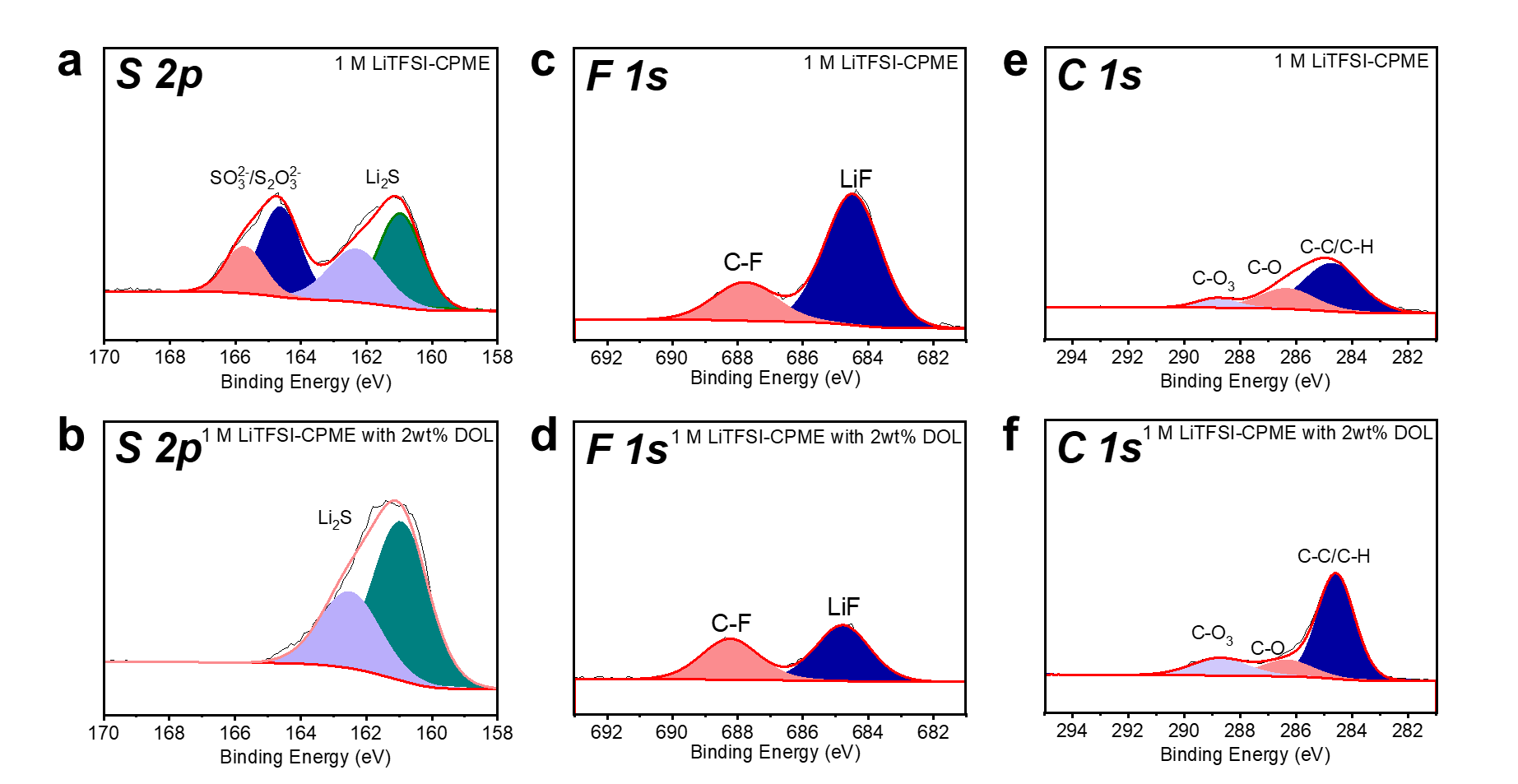


**Figure S16**. XPS of Li metal anode surface after 150 cycles in S/C||Li batteries in 1 M LiTFSI-CPME and 1 M LiTFSI-CPME with 2wt% DOL.
